# Ventral Hernia in the Al-Ahsa Region, Saudi Arabia: Risk Factor Knowledge Assessment

**DOI:** 10.7759/cureus.32581

**Published:** 2022-12-16

**Authors:** Almukhtar Almomatten, Amar A Alonazi, Abdullah M Baragabh, Maitha AlMaghlouth, Ali Alshehri, Mohammed Alessa

**Affiliations:** 1 Medicine, King Faisal University, Al-Ahsa, SAU; 2 Medicine, King Faisal University, Dhahran, SAU; 3 Medicine, King Faisal University, Khobar, SAU; 4 Medicine, King Faisal University, Dammam, SAU; 5 General Surgery, King Faisal University, Al-Ahsa, SAU

**Keywords:** general population, al-ahsa region, risk factors, knowledge, ventral hernia

## Abstract

Introduction

A ventral hernia is defined as a non-inguinal, non-hiatal defect in the fascia of the abdominal wall. Approximately 350,000 ventral hernia procedures are performed each year. Ventral hernia can have a negative impact on a person’s quality of life and, in severe situations, lead to hospitalization and even death.

Aim

This study aimed to assess the knowledge of the general population living in the Al-Ahsa region regarding the risk factors of ventral hernia.

Subjects and methods

This is a cross-sectional study conducted among the general population living in the Al-Ahsa region of Saudi Arabia. A self-administered questionnaire was distributed among the population using an online platform. The questionnaire includes basic demographic characteristics (age, gender, and body mass index (BMI)) and a nine-item questionnaire to assess the knowledge of risk factors of ventral hernia.

Results

Of the 803 respondents involved, 42.1% were aged between 22 and 28 years old, and 44.4% were either overweight or obese. According to participants’ knowledge, the most common risk factor of ventral hernia was heavy weight lifting (87.4%), and pregnancy and labor (64.1%). The overall mean knowledge score was 5.78 (standard deviation (SD): 2.68) out of 12 points. Nearly half (49.4%) were considered to have poor knowledge levels, 40.2% were considered to have moderate knowledge levels, and only 10.6% were considered to have good knowledge levels. Increased knowledge was seen more frequently in younger participants, males, and respondents with normal or underweight BMI.

Conclusion

The knowledge of the general population regarding the risk factors of ventral hernia was insufficient. Of all the population, male respondents who were younger and had a normal body mass index demonstrated a better understanding of the ventral hernia’s risk factors compared to the rest of the subjects. Further research is needed to establish the knowledge of the general population regarding the risk factors of ventral hernia in our region.

## Introduction

A hernia is a condition in which a structure of the body protrudes unnaturally through that normally contains it. Worldwide, it is estimated that more than 20 million hernias are to be repaired yearly. A hernia is divided into three sections: the sac, the neck, and the contents. The most common components are fat and the intestine [1-8]. A ventral hernia is defined as a non-inguinal, non-hiatal defect in the fascia of the abdominal wall. Approximately 350,000 ventral hernia procedures are performed each year [2,3,7].

General surgeons frequently conduct surgery to fix these abnormalities in the abdominal wall with reported 15%-37% and 0.3%-1.4% morbidity and mortality rates, respectively. Surgery is usually suggested for people who have a low operative risk, have symptomatic hernias, or are at a high risk of developing complications. Ventral hernia can have a negative impact on a person’s quality of life and, in severe situations, lead to hospitalization and even death [2,3,7]. In addition, there is a higher chance of complications and recurrence of the hernia postoperatively if the patient does not obtain sufficient medical care [6].

The causes of a ventral hernia can be split into two types: acquired and congenital. Although most hernias seen and treated by general surgeons are acquired, some people are born with ventral hernias and live with them for years before having them surgically repaired. Previous surgery that resulted in an incisional hernia, trauma, and repetitive stress on naturally weak regions of the abdominal wall are all common causes of acquired ventral hernias. The umbilicus, semilunar line, ostomy sites, bilateral inguinal regions, and esophageal hiatus are all naturally occurring weak areas in the abdominal wall. Obesity also plays a role in hernias because it weakens the fascia of the abdomen by stretching it. The action of repeated weight gain and loss causes weakness [4]. In the United States, 348,000 ventral hernia repairs were performed in 2006, with an estimated cost of $3.2 billion [5].

One similar study conducted in the Arar region concluded that abdominal hernias are very common in that region. It also states the significant relationship between obesity and hernias. Lastly, it showed that early diagnosis and health education are important for the prevention of complications [6]. We aim in this study to assess the knowledge of the general population living in the Al-Ahsa region regarding the risk factors of ventral hernia.

## Materials and methods

This is a cross-sectional study conducted among the general population living in the Al-Ahsa region of Saudi Arabia. A self-administered questionnaire was distributed among the population using an online platform during the period between November and December 2021. This study included 803 participants, including 386 males and 417 females aged between 18 and over 35. The questionnaire includes basic demographic characteristics (age, gender, and body mass index (BMI)) and a nine-item questionnaire to assess the knowledge of risk factors of ventral hernia.

The knowledge of participants regarding the risk factors of ventral hernia has been assessed using a nine-item questionnaire, where “yes” coded with 1 and “no/I don’t know” coded with 0 were the answer options. Knowledge question 1 is a four-point Likert scale category with “weak” coded with 1, “fair” coded with 2, “good” coded with 3, and “excellent” coded 4 as the answer options giving a total score points of 12. The total competency score has been calculated by adding all nine items, and a score range from 1 to 12 points has been generated indicating that the greater the score, the greater the knowledge about ventral hernia. Using 50% and 75% as cutoff points to determine the level of knowledge, participants were considered to have poor knowledge if the score was below 50%, 50%-75% were considered moderate knowledge, and above 75% were considered good knowledge levels.

Categorical variables were shown as numbers and percentages (%), while continuous variables were summarized as mean and standard deviation (SD). The differences in the score of knowledge in relation to the sociodemographic characteristics of participants have been performed using Mann-Whitney Z-test. Statistical collinearity has been performed using the Shapiro-Wilk test and the Kolmogorov-Smirnov test. Based on the overall distribution, the knowledge score follows abnormal distribution; thus, the nonparametric test was applied. Two-tailed analyses with p<0.05 were used as the cutoff for statistical significance. All data analyses were performed using the Statistical Package for the Social Sciences (SPSS) version 26 (IBM SPSS Statistics, Armonk, NY, USA).

## Results

This cross-sectional study involved 803 individuals living in Al-Ahsa, Saudi Arabia. As seen in Table 1, 42.1% were aged between 22 and 28 years old with more than half (51.9%) being females. Respondents who were overweight constituted 29.1%, while those who were obese constituted 15.3%.

**Table 1:** Basic demographic characteristics of participants living in Al-Ahsa (N=803) BMI: body mass index

Study data	Number (%)
Age group	
18-21 years	189 (23.5%)
22-28 years	338 (42.1%)
29-35 years	90 (11.2%)
>35 years	186 (23.2%)
Gender	
Male	386 (48.1%)
Female	417 (51.9%)
BMI level	
Underweight (<18.5 kg/m^2^)	67 (8.3%)
Normal (18.5-24.9 kg/m^2^)	379 (47.2%)
Overweight (25-29.9 kg/m^2^)	234 (29.1%)
Obese (≥30 kg/m^2^)	123 (15.3%)

The assessment of the knowledge of ventral hernia was given in Table 2. It can be observed that 43.6% perceived their overall knowledge about hernia as weak. The proportion of respondents who believed that an asthmatic patient has a high chance to develop a hernia was 34.5%, while a greater proportion (87.4%) believed a hernia is related to heavy weight lifting. Also, respondents who believed that hernia is related to constipation and smoking were 55.9% and 28.3%, respectively. Approximately 38% of the respondents thought that patients with an enlarged prostate have a high possibility to suffer from a hernia. Respondents also believed that pregnancy and labor (64.1%), and surgical intervention (56.7%) are related to hernia. Only 23.2% of the respondents believed that diabetic patients have a greater chance of developing a hernia. According to our results, the overall mean knowledge score was 5.78 (SD: 2.68) with poor, moderate, and good levels of knowledge found among 49.4%, 40.2%, and 10.3%, respectively.

**Table 2:** Assessment of the knowledge of ventral hernia (N=803) SD: standard deviation

Statement	Yes (%)
How will you evaluate your knowledge about hernia?	
Weak	350 (43.6%)
Fair	249 (31%)
Good	138 (17.2%)
Excellent	66 (08.2%)
Do you think hernia is related to heavy weight lifting?	702 (87.4%)
Do you think pregnancy and labor can be related to hernia?	515 (64.1%)
Do you think a hernia is related to surgery?	455 (56.7%)
Do you think hernia is related to constipation?	449 (55.9%)
Do you think patients with an enlarged prostate have a high possibility to suffer from a hernia?	305 (38%)
Do you think an asthmatic patient has a high chance to develop a hernia?	277 (34.5%)
Do you think hernia is related to smoking?	227 (28.3%)
Do you think diabetic patients have a high chance to develop hernia?	186 (23.2%)
Total knowledge score (mean±SD)	5.78±2.68
Level of knowledge	
Poor	397 (49.4%)
Moderate	323 (40.2%)
Good	83 (10.3%)

When measuring the differences in the score of knowledge in relation to the basic demographic characteristics of participants (Table 3), it was found that a higher knowledge score was more associated among respondents with younger age (Z=6.711; p<0.001), male gender (Z=2.104; p=0.035), and a normal or underweight body mass index level (Z=3.342; p=0.001).

**Table 3:** Differences in the score of knowledge in relation to the basic demographic characteristics of participants living in Al-Ahsa (N=803) *Statistically significant SD: standard deviation, BMI: body mass index

Factor	Knowledge score (12) (mean±SD)	Z-test	P-value
Age group			
18-28 years	6.25±2.83	6.711	<0.001*
>28 years	4.89±2.11		
Gender			
Male	6.00±2.86	2.104	0.035*
Female	5.58±2.49		
BMI level			
Normal or underweight (<25 kg/m^2^)	6.06±2.71	3.342	0.001*
Overweight or obese (≥25 kg/m^2^)	5.43±2.61		

## Discussion

The present study is carried out to determine the level of knowledge regarding ventral hernia among the general population living in Al-Ahsa, Saudi Arabia. Our results revealed that the general population’s knowledge about the risk factors of ventral hernia was deficient. Of the population, 49.4% were considered to have poor knowledge, 40.2% were considered to have moderate knowledge, and only 10.6% were considered to have good knowledge (mean score: 5.78; SD: 2.68 out of 12 points). These findings are almost consistent with those of Alkhalaf et al. [9]. According to reports, 44.9% of the population living in the eastern region had a poor awareness level regarding hernia, 35.3% had good, 14% had very good, and fewer than 6% demonstrated excellent levels. This is in accordance with the study by Mahfouz et al. [10], wherein 49.1% reported poor awareness levels, while 50.9% had good awareness. Notwithstanding these reports, a study carried out among the general population living in Aljouf found that the knowledge was very good, good, and excellent among 38%, 36%, and 26%, respectively, adding that both males and females have knowledge of the risk factors of hernia regardless of age, gender, and level of education [11]. Although, there were conflicting reports regarding the general population’s knowledge of the disease among English patients who underwent abdominal surgery [12], nearly one-third (30.8%) were not aware that they had an incisional hernia. These patients presented with a smaller hernia and were significantly older. Also, most patients expressed pain associated with discomfort, cosmetic problems, or functional disability. More investigations are warranted to confirm the population’s understanding of ventral hernia and its associated risk factors.

Data in this study indicates that the increase in knowledge was more likely seen in the younger age group (18-28 years), males, and those with normal or underweight BMI. This is in contrast with the paper by Alkhalaf et al. [9], wherein female respondents showed better awareness levels than their male counterparts. In Taif, younger participants exhibited poor knowledge about the disease, and variables such as age, marital status, occupation, number of children, and educational level were also statistically associated with awareness of inguinal hernia [10]. Multicenter studies may be needed to determine the influence of sociodemographic variables in terms of the population’s awareness regarding hernia.

Regarding the perceived general knowledge about hernia, we noted that 43.6% of the subjects perceived their knowledge as weak, 31% as fair, 17.2% as good, and only 8.2% as excellent. Among patients with a hernia who were scheduled for abdominal surgery, their reported knowledge was quite limited, and although their level of understanding about hernia surgery is suboptimal, patients were willing to be urgently operated on or at least be included on the surgical waiting list. The authors further added that there was a long way to go in terms of improving knowledge toward the surgical intervention of hernia [13].

Moreover, we discovered that our population had a good understanding of some of the risk factors of hernia. For example, 87.4% were aware that heavy weight lifting is a risk factor for hernia, 64.1% believed that pregnancy and labor can cause a hernia, and 56.7% and 55.9%, respectively, believed that previous surgery and constipation can lead to a hernia. On the contrary, their awareness of the other risk factors for hernia was deficient, including enlarged prostate (38%) and asthma, smoking, and diabetes (23.2%). These findings are comparable to that of Albukairi et al. [14]. Based on their accounts, 87% of the subjects were of the opinion that there was an association between hernia and heavy lifting, and 65% and 62% were aware that pregnancy and surgery, respectively, were also contributing factors for a hernia, while there was a lack of knowledge that smoking (37%), chronic constipation (36%), prostate enlargement (32%), asthma (32%), and diabetes (29%) were also risk factors for developing a hernia. While there were variations in terms of hernia’s risk factors, however, there were also reports of variations regarding complications after surgical intervention of the hernia. Based on the reports of Ahonen-Siirtola et al. [15], surgical infections, hernia recurrence, and bowel lesions were some of the major complications associated with surgical intervention. This information has to be considered first by patients who intend to undergo intervention and, most of all, to seek advice from a specialist who can provide accurate information and the method for the treatment of the disease.

## Conclusions

The knowledge of the general population regarding the risk factors of ventral hernia was insufficient. Of all the population, male respondents who were younger and had a normal body mass index demonstrated a better understanding of the risk factors of ventral hernia compared to the rest of the subjects. It is necessary to address the gaps in the knowledge. Therefore, extensive efforts are needed to bridge these gaps, and awareness campaigns could be the best method wherein social media and print ad media had vital roles in bringing the information throughout the community. Further research is needed to establish the knowledge of the general population regarding the risk factors of ventral hernia in our region.

## Appendices

Table 4 shows the survey questionnaire that was used to collect basic demographic characteristics (age, gender, and BMI) and a nine-item questionnaire to assess the knowledge of risk factors of ventral hernia.

**Table 4:** Survey

Survey						
Do you agree on participation?	Yes			No		
Participant data						
Do you live in Al-Ahsa?	Yes			No		
Age	18-21	22-28	29-35			More than 35
Gender	Male			Female		
Weight (in kg)						
Height (in cm)						
Assessment of the participants’ knowledge of hernia						
1. How will you evaluate your knowledge about hernia?	0-10 (0 = know nothing, 10 = know everything)					
2. Do you think asthmatic patients have a high chance to develop hernia?	I don’t know	No			Yes	
3. Do you think hernia is related to heavy weight lifting?	I don’t know	No			Yes	
4. Do you think hernia is related to constipation?	I don’t know	No			Yes	
5. Do you think hernia is related to smoking?	I don’t know	No			Yes	
6. Do you think patients with enlarged prostates have a high possibility to suffer from a hernia?	I don’t know	No			Yes	
7. Do you think pregnancy and labor can be related to hernia?	I don’t know	No			Yes	
8. Do you think a hernia is related to surgery?	I don’t know	No			Yes	
9. Do you think diabetic patients have a high chance to develop hernia?	I don’t know	No			Yes	
